# Extraintestinal pathogenic *Escherichia coli *O1:K1:H7/NM from human and avian origin: detection of clonal groups B2 ST95 and D ST59 with different host distribution

**DOI:** 10.1186/1471-2180-9-132

**Published:** 2009-07-07

**Authors:** Azucena Mora, Cecilia López, Ghizlane Dabhi, Miguel Blanco, Jesús E Blanco, María Pilar Alonso, Alexandra Herrera, Rosalía Mamani, Stéphane Bonacorsi, Maryvonne Moulin-Schouleur, Jorge Blanco

**Affiliations:** 1 Laboratorio de Referencia de E. coli, Departamento de Microbiología y Parasitología, Facultad de Veterinaria, Universidad de Santiago de Compostela, Lugo, Spain; 2 Unidad de Microbiología, Complejo Hospitalario Xeral-Calde de Lugo, Spain; 3 Laboratoire d'Études de Génétique Bactérienne dans les Infections de l'Enfant (EA3105), Université Denis Diderot-Paris 7, Service de Microbiologie, Hôpital Robert Debré, Paris, France; 4 Laboratoire de Pathogénie Bactérienne, UR 1282 Infectiologie Animale et Santé Publique, INRA Centre de Tours, Nouzilly, France

## Abstract

**Background:**

Extraintestinal pathogenic *Escherichia coli *(ExPEC) strains of serotype O1:K1:H7/NM are frequently implicated in neonatal meningitis, urinary tract infections and septicemia in humans. They are also commonly isolated from colibacillosis in poultry. Studies to determine the similarities of ExPEC from different origins have indicated that avian strains potentially have zoonotic properties.

**Results:**

A total of 59 ExPEC O1:K1:H7/NM isolates (21 from avian colibacillosis, 15 from human meningitis, and 23 from human urinary tract infection and septicemia) originated from four countries were characterized by phylogenetic PCR grouping, Multilocus Sequence Typing (MLST), Pulsed Field Gel Electrophoresis (PFGE) and genotyping based on several genes known for their association with ExPEC or avian pathogenic *Escherichia coli *(APEC) virulence.

APEC and human ExPEC isolates differed significantly in their assignments to phylogenetic groups, being phylogroup B2 more prevalent among APEC than among human ExPEC (95% vs. 53%, *P *= 0.001), whereas phylogroup D was almost exclusively associated with human ExPEC (47% vs. 5%, *P *= 0.0000). Seven virulence genes showed significant differences, being *fimAv*_MT78 _and *sat *genes linked to human isolates, while *papGII*, *tsh*, *iron*, *cvaC *and *iss *were significantly associated to APEC. By MLST, 39 of 40 ExPEC belonging to phylogroup B2, and 17 of 19 belonging to phylogroup D exhibited the Sequence Types (STs) ST95 and ST59, respectively. Additionally, two novel STs (ST1013 and ST1006) were established. Considering strains sharing the same ST, phylogenetic group, virulence genotype and PFGE cluster to belong to the same subclone, five subclones were detected; one of those grouped six strains of human and animal origin from two countries.

**Conclusion:**

Present results reveal that the clonal group B2 O1:K1:H7/NM ST95, detected in strains of animal and human origin, recovered from different dates and geographic sources, provides evidence that some APEC isolates may act as potential pathogens for humans and, consequently, poultry as a foodborne source, suggesting no host specificity for this type of isolates. A novel and important finding has been the detection of the clonal group D O1:K1:H7/NM ST59 almost exclusively in humans, carrying pathogenic genes linked to the phylogenetic group D. This finding would suggest D O1:K1:H7/NM ST59 as a host specific pathotype for humans.

## Background

Extraintestinal pathogenic *E. coli *(ExPEC) strains are implicated in a large number of infections in humans and animals, such as urinary tract infection (UTI), meningitis, diverse intraabdominal infection, pneumonia, osteomyelitis, and soft-tissue infection; besides, bacteremia can accompany infection at any of these sites. ExPEC, which include avian pathogenic (APEC) *E. coli*, uropathogenic *E. coli *(UPEC), septicemic *E. coli*, and newborn meningitis-causing *E. coli *(NMEC), exhibit considerable genome diversity characterized by the possession of various combinations of adhesins (e.g., P and S fimbriae), iron-acquisition systems (e.g., aerobactin), host defense-avoidance mechanisms (e.g., capsule or O-specific antigen), toxins (e.g., hemolysin), and others (Tsh, IbeA, CNF1, CDT, TraT, etc) which collectively are known as extraintestinal virulence factors [1-3].

APEC strains are responsible for avian colibacillosis in domesticated and wild birds, an illness which starts as a respiratory tract infection and evolves into a systemic infection of internal organs [4,5]. APEC strains show similarities with human ExPEC strains, but it is unclear whether the different ExPEC strains are indistinctly associated with all such invasive diseases in human and animals or whether particular clones are associated with avian colibacillosis, urosepsis or meningitis. The diversity of known and putative ExPEC-associated virulence genes, together with high levels of genetic overlap seen among both pathogenic and non-pathogenic extraintestinal *E. coli *isolates, makes it difficult to attribute a set of factors to a specific group of ExPEC [6]. In fact, different authors have pointed out that there is no unique virulence profile for both UPEC and APEC, emphasizing their potential to be zoonotic agents [7-9].

Among ExPEC strains, the O1 serogroup is one of the most commonly detected in APEC, UPEC, NMEC and septicemic *E. coli *strains [4,7,10-14]. On the other hand, ExPEC strains that cause neonatal meningitis (NMEC) have been typically associated with the K1 capsular antigen [15] and, in the same way, there has been shown a link between APEC strains of serotypes O1:K1, O2:K1, O18:K1 with pathogenicity [7,16]. Ewers et al. [2] found in their study of 526 strains (APEC, UPEC and NMEC), a considerably high number of virulence genes associated with neuC (K1)-positive strains belonging to the three pathogroups.

In the present study, we performed comparative genotyping of APEC, NMEC and septicemic/UPEC isolates belonging exclusively to the proven pathogenic serotype O1:K1:H7/NM, obtained from four countries. The objective was to characterize their content of virulence genes, phylogenetic groups, MLST types and PFGE macrorestriction profiles to better understand the similarities or differences of these ExPEC pathotypes.

## Results and discussion

### Determination of the O:K:H antigens

All 59 isolates included in the present study belonged to the O1:H7 or HNM (nonmotile) serotype, with 24 nonmotile strains. Curiously, 95% (18 of 19) strains belonging to phylogenetic group D showed to be nonmotile against 15% (six of 40) B2 strains (*P *= 0,000). When the isolates were tested by PCR (Table 1) for the presence of the flagellar H7 gene, all but two strains (one B2 and one D) resulted positive. Besides, all 59 isolates showed to possess the *neuC *gene that encodes the K1 capsular antigen.

**Table 1:** ExPEC/APEC genes used for virulence and phylogenetic typing

Category	Gene(s)	Comment	Reference
	*fimH*	D-mannose-specific adhesin, type 1 fimbriae	[7]
	
	*fimAv*_MT78_	Fim A variant MT78 of type 1 fimbriae	[7]
	
	*pap*	Pilus associated with pyelonephritis (P fimbriae)	
	
	*papC*	Pilus assembly; central region of pap operon	[21]
	
	*papG*: *papG *I *papG *II *papG *III	Gal(α 1–4) Gal-specific pilus tip adhesin molecule rare Pyelonephritis-associated Cystitis-associated	[24]
	
Adhesins	*sfa*/*focDE*	Central region of *sfa *(S fimbriae) and *foc *(F1C fimbriae) operons	[21]
	
	*sfaS*	Pilus tip adhesin, S fimbriae (sialic acid-specific)	[7]
	
	*focG*	Pilus tip molecule, F1C fimbriae (sialic acid-specific)	[7]
	
	*afa*/*draBC*	Dr antigen-specific adhesin operons (AFA, Dr, F1845)	[21]
	
	*bmaE*	Blood group M-specific adhesin	[13]
	
	*nfaE*	Nonfimbrial adhesin I assembly and transport	[13]
	
	*gafD*	N-acetyl-D-glucosamine-specific (G, F17c) fimbriae adhesin	[13]

	*cnf*1	Cytotoxic necrotizing factor 1	[7]
	
	*cdtB*	Cytolethal distending toxin	[7]
	
Toxins	*sat*	Secreted autotransporter toxin	[25]
	
	*hlyA*	α- hemolysin	[26]

	*fyuA*	Yersinia siderophore receptor (ferric yersiniabactin uptake)	[13]
	
Siderophores	iutA*	Ferric aerobactin receptor (iron uptake: transport)	[7]
	
	iroN*	Novel catecholate siderophore receptor	[27]

	*neuC*	K1 antigen	[7]
	
	cvaC*	ColV; on plasmids with traT, iss, and antibiotic resistance	[13]
	
Protectin	iss*	Increased serum survival (outer membrane protein)	[27]
	
	traT*	Surface exclusion, serum survival (outer membrane protein)	[13]

	*ibeA*	Invasion of brain endothelium IbeA	[7]
	
	*malX *(PAI)	Pathogenicity-associated island marker	[13]
	
Miscelaneous	*usp*	Uropathogenic-specific protein (bacteriocin)	[28]
	
	*fliCh7*	H7 *fliC *flagellin	[29]
	
	tsh*	Tsh (temperature-sensitive hemagglutinin) serine protease	[7]

Phylogenetic typing	*chuA*	Haem transport gene	[30]
	
	*yjaA*	Gene of unknown fuction from the E. coli K-12 genome	[30]
	
	TSPE4.C2	Anonymous DNA fragment	[30]

*APEC plasmid-associated genes

### Phylogenetic typing

There are several studies suggesting that virulent clonal groups are derived primarily from phylogroup B2, and to a lesser extent from phylogroup D, explaining the predominance of phylogenetic groups B2 and D among clinical isolates [3]. As expected, we found that the 59 ExPEC strains O1:K1:H7/HNM included in this study belonged to the phylogenetic groups B2 and D (68% and 32%, respectively), although significant differences on their association were detected: only one APEC isolate of phylogroup D (5%) against 18 (47%) of human origin (*P *= 0,001) (Table 2).

**Table 2:** Results of genotyping studies in relation to the ExPEC pathotype

	No. of isolates and % prevalence relative to the total (n)			Statistical significance of prevalence *P *value**	
Genetic profile	APEC (n = 21)	NMEC (n = 15)	septicemic /UPEC (n = 23)	APEC vs human ExPEC	septicemic/UPEC vs NMEC

Phylogroup: B2 D	20 (95%) 1 (5%)	6 (40%) 9 (60%)	14 (61%) 9 (39%)	+ (0,001)	- (0,177)

*fimH*	21 (100%)	13 (87%)	20 (87%)	- (0,065)	- (0,370)

*FimAv*_MT78_	2 (10%)	6 (40%)	10 (43%)	+ (0,007)	- (0,551)

*papC*	21 (100%)	13 (87%)	21 (91%)	- (0,162)	- (0,360)

*papG*I	0	1	1	- (0,411)	- (0,491)

*papG *II	21 (100%)	11 (73%)	17 (74%)	+ (0,008)	- (0,291)

*papG *III	0	0	0	-	-

*sfa/focDE*	0	1	0	- (0,644)	- (0,395)

*sfaS*	0	1	0	- (0,644)	- (0,395)

*focG*	0	0	0	-	-

*afa/draBC*	0	0	0	-	-

*bmaE*	0	0	0	-	-

*nfaE*	0	0	0	-	-

*gafD*	0	0	0	-	-

*cnf*1	0	0	0	-	-

*cdt*	0	1	0	- (0,644)	- (0,395)

*sat*	0	10 (66%)	11 (48%)	+ (0,023)	- (0,141)

tsh*	7 (33%)	1 (7%)	2 (9%)	+ (0,018)	- (0,660)

*hlyA*	0	3 (20%)	3 (13%)	- (0,061)	- (0,292)

iroN*	21 (100%)	5 (33%)	10 (43%)	+ (0,000)	- (0,390)

*fyuA*	20 (95%)	15 (100%)	23 (100%)	- (0,356)	- (1,000)

iutA*	20 (95%)	13 (87%)	20 (87%)	- (0,295)	- (0,370)

*neuC *(K1)	21 (100%)	15 (100%)	23 (100%)	-	-

cvaC*	13 (62%)	3 (20%)	6 (26%)	+ (0,003)	- (0,490)

iss*	20 (95%)	3 (20%)	8 (35%)	+ (0,000)	- (0,272)

traT*	20 (95%)	12 (80%)	20 (87%)	- (0,207)	- (0,292)

*malX*	20 (95%)	14 (93%)	23 (100%)	- (0,466)	- (0,395)

*ibeA*	2 (10%)	2 (13%)	1 (4%)	- (0,354)	- (0,286)

*usp*	20 (95%)	14 (93%)	23 (100%)	- (0,466)	- (0,395)

*APEC plasmid-associated genes. Two-way comparisons were performed for each gene and for the phylogroups, using Fisher's exact test. APEC isolates were compared to human ExPEC, and septicemic/UPEC to NMEC. **For each comparison, a *P *value of < 0.05 was considered statistically significant (+), and a *P *value of > 0.05 was not considered statistically significant (-).

In view of the present results, and due to the limited number of avian strains included in the study, we decided to analyze and extra group of 26 APEC isolates O1:K1: [H7]. These new 26 APEC isolates had been originated from different provinces throughout Spain, from 2005 to 2009. By phylogenetic typing, all of them showed to belong to the phylogroup B2, confirming previous results.

### Virulence genotyping

It is difficult a detailed comparison of our results with others' as most studies published concerns more than one serogroup of ExPEC and, consequently, data are not easily comparable. In a recent study, Johnson et al. [17] tested the hypothesis that some APEC strains are a source of human UPEC. For this purpose and after assaying a big collection of more than 1,000 APEC and UPEC strains, the authors chose the APEC O1 (an O1:K1:H7 strain; phylogroup B2) from a mixed cluster with common characteristics (serogroup, phylogenetic group, and virulence genotype) of both APEC and UPEC strains. The authors did not found convincing genetic support for host- or syndrome-specific pathotypes within the broader ExPEC group, based on the provided evidence that the genome sequence of the B2 APEC O1:K1:H7 strain shares strong similarities with some human extraintestinal pathogenic *E. coli *genomes.

In our study, we have found, however, interesting differences. The content of virulence genes was determined by PCR (Table 1) and the results are summarized in Table 2 (in relation to the ExPEC pathotype) and Table 3 (in relation to the phylogenetic group). APEC isolates versus human ExPEC showed statistically significant differences (*P *< 0.05) in seven virulence markers (*fimAv*_MT78_, *papGII*, *sat*, *tsh*, *iroN*, *cvaC *and *iss*), being *fimAv*_MT78 _and *sat *associated with human isolates and, consequently, positively associated with phylogenetic group D; while *papGII*, *tsh*, *iroN*, *cvaC *and *iss *were associated with APEC, resulting *papGII*, *iroN*, *cvaC *and *iss *positively associated with phylogroup B. So, among the six APEC plasmid-associated genes (*cvaC*, *iroN*, *iss*, *iutA*, *traT *and *tsh*) tested, four (*cvaC*, *iroN*, *iss *and *tsh*) were statistically associated with APEC isolates. Three genes (*papGI*, *sat*, *hlyA*) were exclusively detected in isolates of human origin, but only *sat *showed significant differences (*P *= 0,023) with APEC. The other virulence markers analyzed did not show statistical differences, either because they were not detected in any of the 59 isolates (*focG*, *afa/draBC*, *bmaE*, *nfaE*, *gafD*, *cnf*1) or only in one strain (*sfaS*, *cdtB*), or because they were highly prevalent (*fimH*, *papC*, *fyuA*, *iutA*, *traT*, *malX*, *usp*) (*P *> 0.05).

**Table 3:** Results of genotyping studies in relation to the phylogenetic group

	B2 (n = 40)				D (n = 19)				*P *value*
	
Genes	APEC n = 20	NMEC n = 6	Septicemic/UPEC n = 14	TOTAL B2 n = 40	APEC n = 1	NMEC n = 9	UPEC-Sepsis n = 9	TOTAL D n = 19	B2 vs D
***FimAv***_MT78_	2/20(10%)	1/6(16%)	2/14(14%)	5/40(12,5%)	0	5/9(55%)	8/9(89%)	13/19(68%)	+ (0.000)

***papGII***	20/20(100%)	5/6(83%)	14/14(100%)	39/40 (95%)	1/1(100%)	6/9(67%)	3/9 (33%)	10/19(53%)	+ (0.000)

***sat***	0	2/6(33%)	2/14(14%)	4/40(10%)	0	8/9(89%)	9/9(100%)	17/19(89%)	+ (0.000)

***tsh***	6/20(30%)	1/6(17%)	2/14(14%)	9/40(22,5%)	1/1(100%)	0	0	1/19(5%)	- (0.096)

***iro *N**	20/20(100%)	4/6(67%)	10/14(71%)	34/40(50%)	1/1(100%)	1/9(11%)	0	2/19(10,5%)	+ (0.000)

***cva *C**	12/20(60%)	3/6(50%)	6/14(43%)	21/40(52,5%)	1/1(100%)	0	0	1/19(5%)	+ (0.000)

***iss***	19/20(95%)	3/6(50%)	8/14(57%)	30/40(75%)	1/1(100%)	0	0	1/19(5%)	+ (0.000)

Genes showing statistical differences in relation to pathogenic groups were compared for the phylogenetic groups, using Fisher's exact test. *For each comparison, a *P *value of < 0.05 was considered statistically significant (+), and a *P *value of > 0.05 was not considered statistically significant (-).

All the 59 isolates O1:K1:H7/NM showed to accumulate a high number of virulence markers. Thus, 85% of the 40 ExPEC B2 and 74% of the 19 ExPEC D strains were positive for at least eight virulence genes. Twenty-eight different profiles based on the combination of positive virulence genes were observed (Table 4). The 40 isolates belonging to the phylogroup B2 exhibited 19 profiles (1 to 19) with 15 to five virulence genes, and the most prevalent virulence profile was 6–10 detected in 16 isolates of the three ExPEC pathotypes (10 APEC, four UPEC/septicemic *E. coli*, and two NMEC) positive for *fimH*, *papC*, *iroN*, *fyuA*, *iutA*, *cvaC*, *iss*, *traT*, *malX*, and *usp*. The 19 isolates belonging to the phylogroup D exhibited nine profiles (20 to 28) with 10 to five virulence genes, and the most prevalent profile was 21–9 detected in five isolates (three NMEC and two UPEC/septicemic *E. coli*) positive for *fimH*, *fimAv*_MT78_, *papC*, *sat*, *fyuA*, *iutA*, *traT*, *malX*, and *usp*.

**Table 4:** Relationship between virulence genotype and phylogenetic group

B2 (n = 40)			D (n = 19)		
Profile-no. genes*	No. strains	PFGE clusters (no. strains)	Profile-no. genes*	No. strains	PFGE pulsotypes (no. strains)

1–15	1		20-10	1	

2–12	2	III (2) ***subclone A***	21-9	5	II (3) ***subclone E***

3–11	1	VI	22-9	1	

4–11	1	XI	23-8	1	

5–11	1	IV	24-8	4	I (1)

6–10	16	VII(1); VIII(6) ***subclone C***; X(4) ***subclone D***; XI(1); XII(1)	25-8	2	I (1)

7–10	5	IV (3) ***subclone B***; V (1)	26-7	3	

8–10	1		27-7	1	

9-9	1	VI	28-5	1	

10-9	2	VI (2)			

11-9	1	XII			

12-9	1				

13-9	1				

14-7	1	V			

15-6	1	IV			

16-6	1	IX			

17-5	1	VII			

18-5	1	VII			

19-5	1	IX			

*Profile assigned according to the combination and number of virulence genes detected by PCR.

### papG alleles

The *papC *gene was detected in 55 of 59 isolates (93%) (Table 2). Of those 55 *papC *positive isolates, 49 harboured *papG *allele II and two *papG *allele I (one NMEC and one UPEC, both of phylogroup D). The other four positive *papC E. coli *were negative for all three *papG *alleles (one NMEC and three UPEC/septicemic *E. coli*, all of phylogroup D). These four strains were tested again by PCR with primers designed by us to check if they possessed new *papG *varieties. The results showed that the four strains possessed a truncated *pap *operon (data not shown).

### Characterization of ExPEC isolates by MLST

Multilocus sequence typing (MLST) is a DNA sequence-based method that has become of reference to characterize *E. coli *clones. It has been used to study the population biology of pathogenic microorganisms including *E. coli *[18], so that the genetic relatedness between isolates can be compared and closely related organisms can be grouped as clonal complexes.

**Figure 1:**
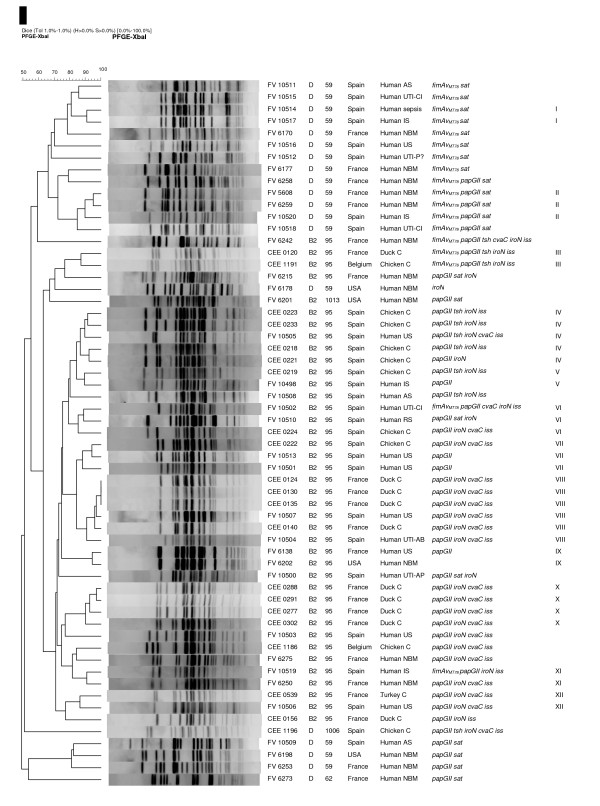
**Pulsed field gel electrophoresis of XbaI-digested DNA from the 59 ExPEC strains included in the study**. Strain designation, phylogenetic group, ST assignation, clinical and geographical origin of isolation, PFGE cluster (>85% similarity), and PCR result for virulence genes that exhibited significant differences within the pathogenic groups are shown at right. This unweighted pair-group method with arithmetic mean dendrogram was generated in BioNumerics software (Applied Maths, St-Martens-Latem, Belgium) by using Dice coefficient with a 1.0% band position tolerance. The scale above the dendrogram indicates percent similarity. AS: abdominal sepsis; UTI: urinary tract infection; CI: cystitis; IS: intestinal sepsis; NBM: Newborn Meningitis; US: urosepsis; P?: posible pyelonephritis; C: colibacillosis; RS: respiratory sepsis; AP: acute pyelonephritis; AB: asymptomatic bacteriuria.

ST95 complex has been reported to contain the related bacteria of serogroups O1, O2 and O18 that express the K1 polysaccharide [14,18,19]. Lau et al. [20] also detected ST59 complex in one O1 isolated. In the present study, MLST analysis of the 59 ExPEC strains O1:K1:H7/NM identified those two ST complexes and five different STs with the same combination of alleles across the seven sequenced loci: ST95 (39 strains-phylogroup B2), ST59 (17 strains-phylogroup D), ST62 (one strain-phylogroup D), and two novel combination of alleles that were assigned to the new ST1006 (one strain-phylogroup D) and ST1013 (one strain-phylogroup B2) (Figure 1).

### Macrorestriction profiles by PFGE

Figure 1 shows a dendrogram with the XbaI macrorestriction profiles obtained by PFGE of the 59 ExPEC strains analyzed. As expected, strains of the same phylogenetic group and ST clustered together (all but one strain, FV 6178 D ST59). Thirty-nine of 40 strains belonging to phylogenetic group B2 constituted one large cluster (63% similarity) which enclosed 38 ST95 B2 strains, one ST1013 B2 strain, and one ST59 D strain. The remaining ST95 B2 strain (FV 6259) was placed close to the large cluster, but with a similarity of 55%. The 39 B2 strains, grouped in the large cluster of 63% similarity, enclosed ten small subclusters of similarity >85% (III to XII). By contrast, strains of the phylogroup D showed by PFGE to be more heterogeneous than those of phylogroup B2. Thus, 18 of the 19 strains belonging to phylogroup D were separately grouped at both extremes of the dendrogram; with one cluster of 13 ST59 D strains, all positive for *fimAv*_MT78 _and *sat *genes at one end (66% similarity); and the remaining five D strains constituting an heterogeneous group at the other end of the dendrogram. Strains of the phylogenetic group D formed only two small subclusters of similarity >85% (I and II).

In a similar study, Moulin-Schouleur et al. [16] comparing O18:K1:H7 isolates of human and avian origin did not detect PFGE profiles with an identity higher than 80% between avian and human ExPEC strains. By contrast, in the present study, PFGE revealed 12 clusters of 85% similarity (I to XII) grouping 36 (61%) of 59 strains, with clusters IV, V, VI, VII, VIII and XII including APEC and human UPEC/septicemic strains (all belonging to the clonal group B2 ST95).

In view of the results obtained in the present study by phylogenetic typing and MLST, two clonal groups (ST95 B2 and ST59 D) could be defined among pathogenic ExPEC strains of the serotype O1:K1:H7/HNM. The ST95 B2 isolates constitute a homogeneous clonal group on the basis of the considerable similarity of the PFGE profiles that indicates recent divergence from a common ancestor. Furthermore, if we consider strains sharing the same ST, the same phylogenetic group, the same PFGE cluster and the same virulence genotype to belong to the same subclone, four closely related subclones were defined among strains ST95 (Figure 1; Table 4): subclone A (two strains B2, cluster III, genotype 2–12); subclone B (three strains B2, cluster IV, genotype 7–10); subclone C (six trains B2, cluster VIII, genotype 6–10); and subclone D (four strains B2, cluster X, genotype 6–10). Interestingly, subclone C grouped six strains (two of human and four of animal origins) originated from two different countries. On the other hand, strains belonging to the clonal group D ST59 (17 isolates among those 19 of phylogroup D), showed very specific characteristics, different from those of phylogenetic group B2. Thus, D O1:K1:H7/NM ST59 strains were almost exclusively isolated from humans; all but one showed to be nonmotile (HNM), versus six of 40 B2 strains (*P *= 0,000); the virulence gene profile of these strains was also different, in fact the nine profiles exhibited by group D were exclusive of it; and genes *fimAv*_MT78_, *sat *were significantly linked to this phylogroup (detected in 13 and 17 strains, respectively). By PFGE, D O1:K1:H7/NM ST59 strains showed to be very heterogeneous. Thus, 16 of 17 ST59 appeared grouped in two separated clusters of 66 and 81% similarity, respectively. Only one subclone sharing the same ST, phylogenetic group, PFGE cluster and virulence genotype was identified: subclone E (three strains D, cluster II; genotype 21-9).

## Conclusion

As shown in previous studies, some closely related clones can be involved in extraintestinal infections in humans and poultry [7,8,16,17]. Most of these studies included strains of various serogroups, so it is difficult a detailed comparison to know whether APEC and human strains are identical or not. In order to answer this question, we focused our work on a collection of avian and human ExPEC strains belonging exclusively to the serotype O1:K1:H7/NM which is one of the predominant serotypes implicated in neonatal meningitis, UTI, septicemia, as well as in avian collibacilosis.

Some interesting remarks can be posed from our study. Firstly, we have detected a high prevalence of genes known for their association with ExPEC or APEC virulence (81% of 59 isolates showed to be positive for at least eight virulence genes), confirming the pathogenic potential of O1:K1:H7/NM strains. Besides, we have detected significant genetic differences translated into two clonal groups defined on the basis of phylogenetic typing and MLST: B2 ST95 O1:K1:H7/NM and D ST59 O1:K1:H7/NM. The clonal group B2 ST95 detected in APEC and human ExPEC strains, recovered from different dates and geographic sources (four countries; from 1988 to 2003) provides evidence that some APEC isolates may act as potential pathogens for humans and, consequently, poultry as a foodborne source, suggesting no host specificity for this type of isolates. Finally, a novel and important finding in our study has been the detection of the clonal group D O1:K1:H7/NM ST59 strains exclusively in humans (17 strains, in three countries, 1988 to 2002), carrying pathogenic genes linked to the phylogenetic group D, which would suggest a host specific pathotype. Due to the limited number of avian strains included in the study, and in view of the importance of this conclusion, we analyzed and extra group of 26 APEC isolates O1:K1: [H7] from different provinces throughout Spain, obtained from 2005 to 2009. By phylogenetic typing, all of them showed to belong to the phylogroup B2, confirming previous results. Further research is necessary to deeply analyze this clonal group apparently specific of human isolates.

## Methods

### Bacterial isolates

A total of 59 extraintestinal pathogenic *E. coli *(ExPEC) from veterinary and medical origins were used in this study. All the isolates analyzed here belonged to the serotype O1:K1:H7/NM and were obtained from 1988 to 2003 in previously described studies [10-12,21,22]. Twenty-one ExPEC were isolated from avian colibacillosis (APEC isolates = 10 chicken, 10 duck, and one turkey) in Belgium, France, and Spain; 15 isolates were obtained from human meningitis (NMEC isolates) in France, and USA; and 23 ExPEC were isolated from human cases of UTI and sepsis in Spain (UPEC/septicemic *E. coli *isolates). Strains were stored at room temperature in nutrient broth (Difco) with 0.75% of agar.

### Serotyping

The determination of O and H antigens was carried out using the method previously described by Guinée et al. [23] with all available O (O1 to O181) and H (H1 to H56) antisera.

The presence of the capsular antigen K1 was detected by amplification of the *neuC *gene. Additionally, all strains were tested by PCR to detect the presence of the flagellar H7 gene (Table 1) [24-30].

### Phylogenetic analysis and virulence genotyping

Isolates were assigned to one of the four main phylogenetic groups of *E. coli *(A, B1, B2 and D) by using the multiplex PCR-based method of Clermont et al. [30].

For virulence typing, all isolates were screened by PCR amplification for the presence of several genes known for their association with ExPEC or APEC virulence: *fimH*, *fimAv*_MT78_, *papC *(positive results were tested for *papG *I, *papG *II, *papG *III alleles), *sfa *and *foc *(were analyzed together and positive results were tested for *sfaS *and *focG*), *afa/draBC*, *bmaE*, *nfaE*, *gafD*, *cnf*1, *cdtB *(positive results were tested for *cdt*1, *cdt*2, *cdt*3, *cdt*4 alleles), *sat*, *tsh*, *hlyA*, *iroN*, *fyuA*, *iutA*, *neuC*, *cvaC*, *iss*, *traT*, *malX*, *ibeA*, *usp*. Amplification procedures have been documented elsewhere [7,13,21,24-30] (Table 1).

### MLST

Multilocus sequence typing (MLST) was carried out as previously described [18]. Gene amplification and sequencing of the seven housekeeping genes (*adk*, *fumC*, *gyrB*, *icd*, *mdh*, *purA*, and *recA*) were performed by using the primers and protocol specified at the *E. coli *MLST web site http://mlst.ucc.ie/mlst/dbs/Ecoli. Sequences were reviewed by visual inspection with BioEdit Sequence Alignment Editor (version 7.0.9; Ibis Biosciences). The ClustalW2 program was used to align the sequences. The allelic profile of the seven gene sequences, the Sequence Types (STs), as well as the Sequence complexes (defined as STs that are linked by distances of one or two allelic differences) were obtained via the electronic database at the *E. coli *MLST web site.

### Sequencing

The nucleotide sequence of the amplification products purified with a QIAquick DNA purification kit (Qiagen) was determined by the dideoxynucleotide triphosphate chain-termination method of Sanger, with the BigDye Terminator v3.1 Cycle Sequencing Kit and an ABI 3100 Genetic Analyzer (Applied Bio-Systems).

### Pulse Field Gel Electrophoresis (PFGE)

Cleavage of the agarose-embedded DNA was achieved with 0.2 U/μl XbaI (Roche) according to instructions of the manufacturer. XbaI-digested genomic DNA was analyzed in 1% agarose gel in 0.5× Tris-boric acid-EDTA TBE buffer at 14°C by using CHEF MAPPER (BioRad). The runtime was 21.30 h at 6 V/cm, with initial and final switch times of 2.16 and 54.17 s, respectively. The gel was stained with ethidium bromide (1 μg/mL), observed on the Gel Doc 2000 system (BioRad), and analyzed with the BioNumerics fingerprinting software (Applied Maths, St-Martens-Latem, Belgium). Cluster analysis of the Dice similarity indices based on the unweighted pair group method using arithmetic averages (UPGMA) was done to generate a dendrogram describing the relationship among PFGE profiles. Isolates were considered to be related if their Dice similarity index was > 85% according to Tenover's criteria (≤ six bans of difference) [31].

### Statistical analysis

For APEC, NMEC and septicemic/UPEC populations, Fisher's exact test was used to test the null hypothesis of equal gene prevalence rates across the three populations studied. For each comparison, a *P *value of < 0.05 was considered to denote significant differences.

## Authors' contributions

AM carried out the MLST studies, the analysis and interpretation of all data, and drafted the manuscript. CL carried out the PFGE studies and participated, together with GD, AH and RM in the genotyping of the isolates. MB has made substantial contributions in the design of the PCR and genotyping studies. JEB is responsible of the serotyping. MP carried out the partial characterization of the Spanish human isolates. SB and MM contributed with the partial characterization of human and APEC isolates from other countries, respectively. JB conceived the study, participated in its design and, together with AM, drafted the manuscript. All authors read and approved the final manuscript.
